# Prediction of Colon Cancer Stages and Survival Period with Machine Learning Approach

**DOI:** 10.3390/cancers11122007

**Published:** 2019-12-12

**Authors:** Pushpanjali Gupta, Sum-Fu Chiang, Prasan Kumar Sahoo, Suvendu Kumar Mohapatra, Jeng-Fu You, Djeane Debora Onthoni, Hsin-Yuan Hung, Jy-Ming Chiang, Yenlin Huang, Wen-Sy Tsai

**Affiliations:** 1 Department of Computer Science and Information Engineering, Chang Gung University, Guishan 33302, Taiwan; d0521006@cgu.edu.tw (P.G.); d0421008@cgu.edu.tw (D.D.O.); 2 Division of Colon and Rectal Surgery, Chang Gung Memorial Hospital, Linkou 33305, Taiwanhsinyuan@cloud.cgmh.org.tw (H.-Y.H.); jmjiang1234@yahoo.com.tw (J.-M.C.);; 3 Graduate Institute of Clinical Medical Sciences, College of Medicine, Chang Gung University, Guishan 33302, Taiwan; 4 Volktek Corporation, New Taipei 23553, Taiwan; suvendu.mohapatra@volktek.com; 5 College of Medicine, Chang Gung University, Guishan 33302, Taiwan; dochempath@cgmh.org.tw; 6 Department of Anatomic Pathology, Chang Gung Memorial Hospital, Linkou 33305, Taiwan

**Keywords:** colon cancer, artificial intelligence, machine learning, TNM staging, disease-free survival, prediction

## Abstract

The prediction of tumor in the TNM staging (tumor, node, and metastasis) stage of colon cancer using the most influential histopathology parameters and to predict the five years disease-free survival (DFS) period using machine learning (ML) in clinical research have been studied here. From the colorectal cancer (CRC) registry of Chang Gung Memorial Hospital, Linkou, Taiwan, 4021 patients were selected for the analysis. Various ML algorithms were applied for the tumor stage prediction of the colon cancer by considering the Tumor Aggression Score (TAS) as a prognostic factor. Performances of different ML algorithms were evaluated using five-fold cross-validation, which is an effective way of the model validation. The accuracy achieved by the algorithms taking both cases of standard TNM staging and TNM staging with the Tumor Aggression Score was determined. It was observed that the Random Forest model achieved an F-measure of 0.89, when the Tumor Aggression Score was considered as an attribute along with the standard attributes normally used for the TNM stage prediction. We also found that the Random Forest algorithm outperformed all other algorithms, with an accuracy of approximately 84% and an area under the curve (AUC) of 0.82 ± 0.10 for predicting the five years DFS.

## 1. Introduction

According to the World Cancer Research Fund, colorectal cancer is the third most common cancer worldwide, which has affected over 1.8 million new patients in 2018 [1]. According to a report issued by the Health Promotion Administration, Taiwan, colorectal cancer topped at the list of most commonly occurring cancer in 2016 [2]. Based on the death rate, colorectal cancer is the second leading cause of death in the United States, where the number of deaths was 14.2 per 100,000 men and women per year based on 2012–2016 deaths statistics [3]. In Taiwan, colorectal cancer ranked as the third leading cause of mortality due to cancer in 2016. There were 47,760 deaths due to cancer, which accounted for 27.7% of the total deaths in 2016. The crude death rate for CRC was 24.3 deaths per 100,000 populations, an increase of 0.1% compared to the previous year; the age-adjusted death rate was 14.6 deaths per 100,000 populations, a decrease of 0.3%, which resulted in 12% of all cancer-related deaths in 2016. In addition, it was observed that the mortality in men (28.1 per 100,000) was higher as compared to the women (20.6 per 100,000) [4]. In a study made by the American Cancer Society [5], it is predicted that the risk of colon cancer will be increased by 90% and 27.7% for persons aged 20–34 and 35–49, respectively due to the changing lifestyle of individuals.

Various parameters are collected for each colorectal cancer patient such as gender, age, body mass index (BMI), family history (FH), smoking habit, alcohol consumption, and pathological finding including the tumor size, tumor differentiation, circumferential involvement, tumor stage, node stage etc. Several studies considered the tumor size as an important factor for determining the stage of colon cancer [6,7,8,9], whereas some studies focused on evaluating the tumor size as a prognostic factor for the colorectal cancer prognosis [10,11]. It has also been found that the tumor size is not only an essential factor in determining the TNM stage of the colon cancer; it is also a significant predictor, which can be used for estimating the survival period of the patients [12]. The authors in [13] discussed the association of smaller tumor size with higher cancer-specific survival in case of colon cancer. In recent years, the use of machine learning has gained much importance in the field of clinical study [14,15]. Recently, many researchers and clinicians have proposed the use of machine learning in the field of colorectal cancer [16,17,18] and other types of cancers [19,20,21,22,23,24]. For instance, in [25] authors used ML for prostate cancer screening and have determined the impact of few variables such as rate of change of prostate-specific antigen (PSA), age, BMI, and race on the model’s accuracy. They found that inclusion of PSA and age could increase the accuracy by 6%, though the age and race had minimal effect.

The use of machine learning, which can be applied using supervised or unsupervised approaches, has increased tremendously in the field of clinical research. In case of supervised approach of machine learning, the output classes to be predicted are known to us, which are mostly used in the medical research. In [26], authors used age, carcinoembryonic antigen (CEA) test results, tumor location along with standard TNM staging system. It was found that their methods achieved better accuracy over the standard TNM staging system. However, to the best of our knowledge, none of the works published so far have considered the use of tumor related histopathological parameters such as circumferential involvement and tumor grade, along with standard TNM staging parameters for expanding the TNM staging system. As a result, we planned to achieve this objective by analyzing the colon cancer patients’ data to develop a predictive model for the colon cancer tumor stage prediction and survival period prediction using the tumor-related histo-pathology parameters as the prognostic factors. We proposed here the use of Tumor Aggression Score (TAS), which is the combination of tumor size, circumferential involvement, and tumor differentiation for determining the tumor stages. It is to be noted that the Primary Tumor is categorized as TX, T0, Tis, T1, T2, T3, T4a, and T4b according to the cancer staging manual of the American Joint Committee on Cancer (AJCC). In this work, only T1, T2, T3, and T4 categories of the Primary Tumor are considered for the staging. Henceforth, T1, T2, T3, and T4 categories of the Primary Tumor are referred to as the Tumor stage (T-stage) throughout this paper. Consequently, we hypothesized that the circumferential involvement and histological grade would be associated with the prediction of the tumor stages of the cancer along with the tumor size. To address this hypothesis, we analyzed 4021 patients from the CRC department of Chang Gung Memorial Hospital, Linkou, Taiwan.

We used different machine learning classifiers such as Random Forest, Support Vector Machine, Logistic Regression, Multilayer Perceptron, K-Nearest Neighbors, and Adaptive Boosting. These classifiers were trained using supervised learning approach and performances of the models were evaluated using different metrics such as accuracy, precision, recall, and F-measure. We used the Scikit-learn [27] 0.19.2 version and Python 3.5.2 with TensorFlow 1.12 as our backend for the analysis. The collected data were preprocessed by deleting the missing values and were normalized for the remaining data. The results obtained for the colon cancer tumor staging and survival prediction are discussed in the later sections of our article. In the final section of our article, concluding remarks are made along with the future directions of this study.

## 2. Materials and Methods

**Figure 1:**
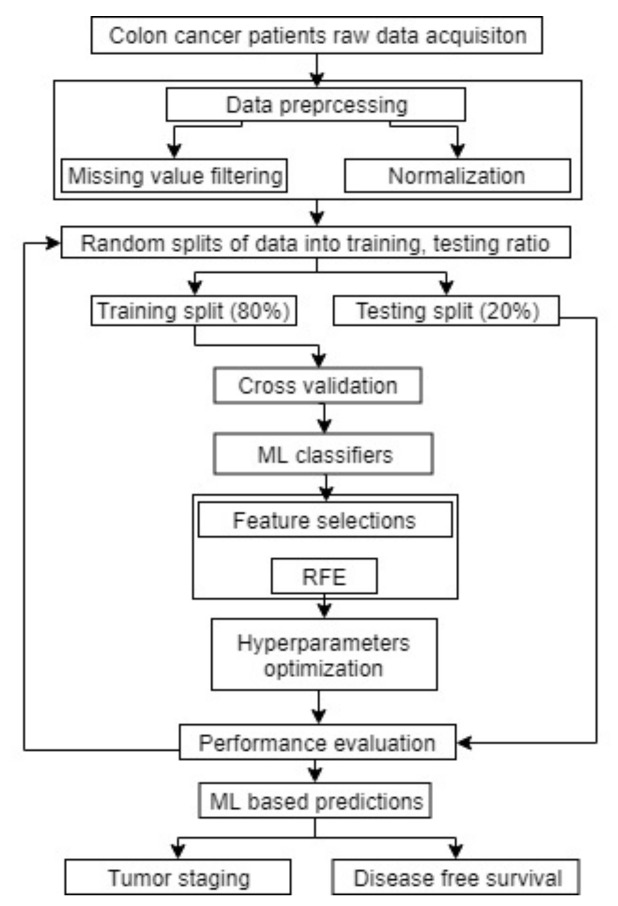
Outline of colon cancer staging and survival prediction using machine learning.

In this section, we described our data acquisition methods along with the approval of the data collection details. We also discussed the data preprocessing and feature selection methods. Furthermore, we discussed about the tumor-related histopathology parameters for creating a new parameter named as TAS. Outline of our study is shown in Figure 1.

### 2.1. Data Acquisition

Our investigation consisted of 4021 patients’ data that were diagnosed with colon cancer. The study was approved by the Institutional Review Board (IRB)/Ethical Committee of Chang Gung Memorial Hospital, Linkou, Taiwan, under the license number 201702073B0. The available clinical and pathological parameters of each patient diagnosed with colon cancer were reviewed. The detailed personal information about the patients was anonymized, and the CRC-related variables were retrieved from the Registry of the Colorectal Cancer in Chang Gung Memorial Hospital, Linkou, Taiwan. All parameters were obtained from different sources of the clinical system as shown in Table 1.

**Figure 2:**
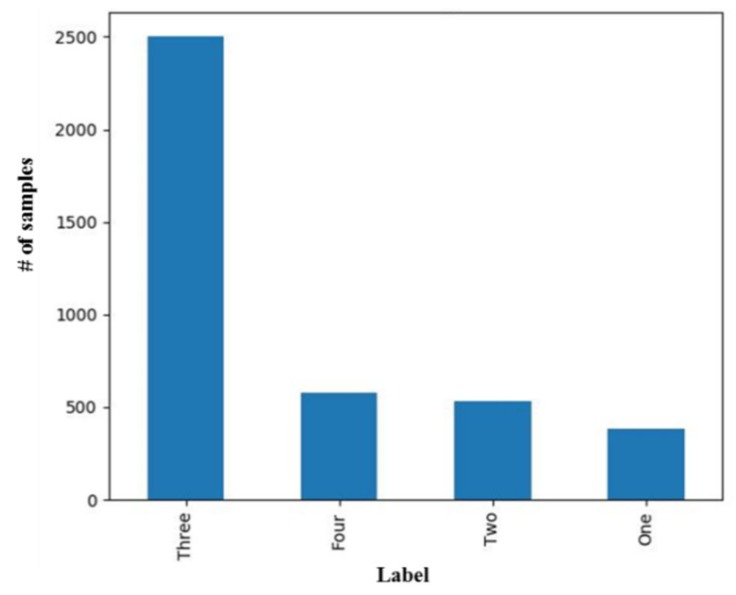
Distribution of patients based on the T-Stage.

As presented in Table 2, the parameters included in the study were: body mass index (BMI) that was categorized as <18.5, underweight; 18.5–23.9, normal; 24.0–26.9, pre-obesity; and exceeding 27, leads to obesity; family history that described if the patient’s immediate relative had some cancer or another hereditary disease; age that was classified as <50; and ≥50; and gender. The history of the hypertension, diabetes, and personal habits such as smoking and alcohol consumption were also collected for the patient. The preoperative carcinoembryonic antigen (CEA) level (normal <5 ng/mL and abnormal ≥5 ng/mL), hemoglobin level (not normal: <11 g/dL and normal: ≥11 g/dL), albumin level (LAB_ALB) (normal <3.5 g/dL and abnormal ≥ 3.5 g/dL), creatinine level (LAB_CR), and white blood cells (WBC) count were also recorded. These parameters were collected using the blood samples obtained from the patients’ pre-operation and follow up procedure. The histopathology parameters of the patients such as the tumor length (Tumrlen), tumor width (Tumrwid), circumferential involvement (CirInvo), tumor differentiation (TumrDiff), tumor stage (T stage), and node stage (N stage) were also recorded. These histopathology parameters were collected after a patient went for the biopsy or surgery. The *p*-value for each parameter was also determined using Chi2 method to understand the importance of the collected parameters. The Chi2 test measured the sample and target vectors as the input and returned the Chi2 statistics and *p*-value of each feature. The data comprised of more number of patients belonging to the tumor stage T3 as compared to other stages, as shown in Figure 2, where label one, two, three, and four represents the tumor stage T1, T2, T3, and T4, respectively.

### 2.2. Data Preprocessing

Before proceeding further for the analysis with 4021 patients’ information, preprocessing of the data was required. In our study, 4003 patients’ instances were completed with no missing values out of 4021 patients’ instances, whereas some patients’ instances such as the values of circumferential involvement, smoking history, and tumor differentiation had some missing values, which were removed. In addition, the collected data had different ranges for the values of different parameters. Therefore, the data were normalized using Scikit-learn.

### 2.3. Derivation of Tumor Aggression Score (TAS)

Based on our hypothesis, we assumed that the tumor-related histopathological parameters were influential parameters, which could be used for the prediction of the tumor stage of the colon cancer. The tumor specimen was inspected and measured by the pathologists after formalin fixation. The tumor size was recorded as ‘length’ times ‘width’. During the pathological examination, the colon was often cut open longitudinally. The tumor ‘length’ means the ‘longitudinal extent’ in centimeter, while the tumor ‘width’ means the ‘transverse extent’ vertical to the tumor length in centimeter. All these parameters were recorded following the pathological reports. Although the tumor size was collected considering the tumor length and tumor width, the maximum values between the tumor length and tumor width were considered for our analysis. Therefore, the maximum size of the tumor (T_max_) was determined using Equation (1) as follows:

T_max_ = max (t_len_, t_wid_) (1)

where, t_len_ and t_wid_ represents the Tumrlen and Tumrwid, respectively. Another influential parameter related to the tumor, cirInvo usually shows the invasion of tumor towards the wall. Similarly, if the tumor is well-differentiated, it increases the chances of detection and vice-versa. When all the influential parameters were determined, Equation (2) was developed considering only those tumor-related parameters as given below:

TAS = T_max_ + C_invo_ + T_dif_ (2)

where, C_invo_ and T_dif_ represents the circumferential involvement and tumor differentiation, respectively. Equation (2) was used to train the system using ML algorithms in Scikit-learn.

### 2.4. Machine Learning Analysis

The new attribute TAS is created as a prognostic factor to investigate its influence on the TNM staging. Hence, it was necessary to evaluate the performance of that attribute for predicting the tumor stage and DFS period of the colon cancer patients. The data considered in our study consist of multiple features and large number of instances. It is difficult to correlate the features manually and predict the outcome of a patient in terms of tumor staging or DFS period. Therefore, we used different ML classifiers such as Random Forest, Support Vector Machine (SVM), Logistic Regression (LR), Multilayer Perceptrons (MLP), K-Nearest Neighbors (KNN), and Adaptive Boosting (AdaBoost) [22,23,24,28], which are popularly used in medical data analysis. Implementing different classifiers in Scikit-learn for the multi-class classification, the scheme of one-against-one was used for the SVM and the scheme OvR was used for Logistic Regression (LR) and other models, which gave the average of all metrics used in our analysis. In total, 4003 samples were used for the analysis. In each round of the experiment, the data were randomly split into 80% for the training of the ML models using five-fold cross-validation and 20% was reserved for the independent testing. During the cross-validation, recursive feature elimination (RFE) method was carried out for the feature selections within the cross-validation. The RFE method removes the weakest features, while attempting to eliminate the dependencies and collinearity that may exist in the model. In addition, there are many hyperparameters associated with each ML model and decision on the values of hyperparameters is very important. Some of the important hyperparameters are discussed here.

In Random Forest, the number of estimators to create the number of decision trees and split the selection criteria to measure the quality of splits needs to be considered. In the case of SVM and LR, the multi-class setting to “OvR” and maximum number of iterations are important. In the case of LR, the use of “solver” as an optimizer is also important. In the case of KNN, the number of neighbors and weights of the points in the neighborhood are important. In the case of Multilayer Perceptrons (MLP), the number of hidden layers, activation function, “solver” as an optimizer, batch size, learning rate, and maximum number of iterations are important. The base estimator, learning rate, number of iterations and the boosting algorithm to be used are important in AdaBoost. In our analysis, we used Scikit-Optimize (skopt) library for automatic optimization of the hyperparameters. The actual settings of some of the best values of the hyperparameters provided by the library for the experiment performed in Section 3.2 were found to be different for every model. For instance, in the case of Random Forest, our best parameters values were number of estimators = 702, maximum depth = 5, minimum samples for split = 2, and minimum samples for leaf = 1. In the case of SVM, the best parameters values were Coefficient = 0.60, degree = 9, tolerance = 0.857947, maximum iteration = 8473. In case of Logistic Regression, the hyperparameters were tolerance = 1.000000, maximum iteration = 13. The MLP had the best parameters setting for the learning rate = 0.012, hidden layer = 934, tolerance = 0.0001, and maximum iteration = 1677. In the case of KNN, the best parameter setting was for the number of neighbors = 14, leaf size = 99. Finally for the AdaBoost Classifier, the best value for the learning rate was found to be 0.131607, and number of estimators was found to be 208.

## 3. Results

We investigated the role of tumor-related histopathology parameters such as CirInvo, TumrDiff, Tumrlen, and Tumrwid for predicting the tumor stage of colon cancer and ultimately to find the relationship between the TAS and five years DFS. It is to be noted that parameters such as only tumor size, N stage (lymph node status), and metastasis are considered in the standard TNM staging method. However, a new score named as TAS was derived in our work based on the hypothesis of using four tumor-related parameters as given in Equation (2). The five-fold cross-validation was performed for evaluating the robustness of different models based on the different testing sets used in each fold to prevent the over-fitting problem. The models were evaluated using an independent testing set. The whole datasets were split into training and testing randomly, where training sets were used for feature selection within fivefold cross-validation. The average values obtained in the model derivation using fivefold cross-validation was recorded along with the final testing results obtained from the independent tests. During model derivation, the feature selection method was also performed within cross-validation to prevent the over-fitting. The creation of the models using the feature selection and cross-validation was referred to as the “training models”, where the settings of hyperparameters vary. The finally derived model that was obtained after getting the best hyper-parameter values was decided by the machine and was referred to as the “testing models”. All implementations were carried out using GPU version of TensorFlow 1.12 with the specification GeForce GTX 1070 Ti, Intel Core (TM) i7-8700k, 3.7 GHz processor, with 32 GB memory, Nvidia-smi 418 in Ubuntu 18.04 platform.

The average performances of different algorithms were measured in terms of the number of instances correctly classified as the required-true positives (TP) and the number of instances incorrectly classified as the required-false positives (FP). The numbers of instances correctly classified as not required are true negatives (TN), and the number of instances incorrectly classified as not required are the false negatives (FN). Using TP, FP, TN, and FN, different values of precision, recall, accuracy, and F-measure were obtained using the following equations, respectively.

Precision = TP/(TP + FP),(3)

Recall = TP/(TP + FN),(4)

Accuracy = TP + TN/(TP + FP + TN + FN),(5)

F-measure = 2 (Precision × Recall)/(Precision + Recall),(6)

where, the F-measure is the weighted harmonic mean of the precision and recall representing the overall performance. 

### 3.1. Prediction of Tumor Stage with Tumor Size as a Prognostic Factor

The current standard of the TNM staging prediction includes the tumor stage, node stage, and metastasis stage. The tumor stage, node stage, and metastasis stage are predicted based on the tumor size, number of affected lymph nodes and different clinical and other imaging tests, respectively. Based on the standard method of the TNM staging, we considered only tumor size as the prognostic factor for predicting the tumor stage of the colon cancer and our observations are recorded in Table 3, as follows.

If tumor size only was considered as a prognostic factor, the models like Random Forest and Adaptive Boosting performed well with accuracy of 0.73 and F-measure of 0.67 approximately as compared to other models. However, the performance was not good enough to be considered for the clinical research. When sensitivity of the model was considered in differentiating the tumor stage, the Random Forest achieved the sensitivity of 0.74, as shown in Table 4, which was better than all other models. Nonetheless, such low sensitivity is not acceptable in clinical practice as further treatment strategies are determined based on the detected stages.

### 3.2. Prediction of Tumor Stage with TAS as a Prognostic Factor

Upon performing the first round of the experiment based on our hypothesis, we created a new variable “Tumor Aggression Score (TAS)” that represents the extent and structure of the tumor growth. We considered this variable as a prognostic factor for predicting the tumor stage. In order to verify the correctness of our assumption, we performed another round of experiment considering TAS as a variable, the results of which are presented in Table 5.

As we know, the TNM stage of the colon cancer patients depends on the tumor stage, which must be determined correctly. Based on the results of the ML models derived using TAS, it can be clearly observed from the Table 5 that not only the tumor size, but also the circular involvement and tumor grade are useful to determine the tumor stage. The TAS adds more importance and sensitivity to the correct determination of the tumor stage, which ultimately benefits the TNM staging. It is evidently observed from the Table 6, where the derived model was able to determine the correct tumor stage with an accuracy of 0.89 and sensitivity of 0.88 as achieved by the Random Forest.

**Figure 3:**
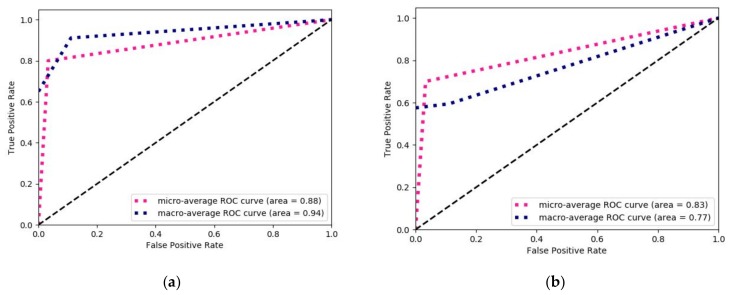

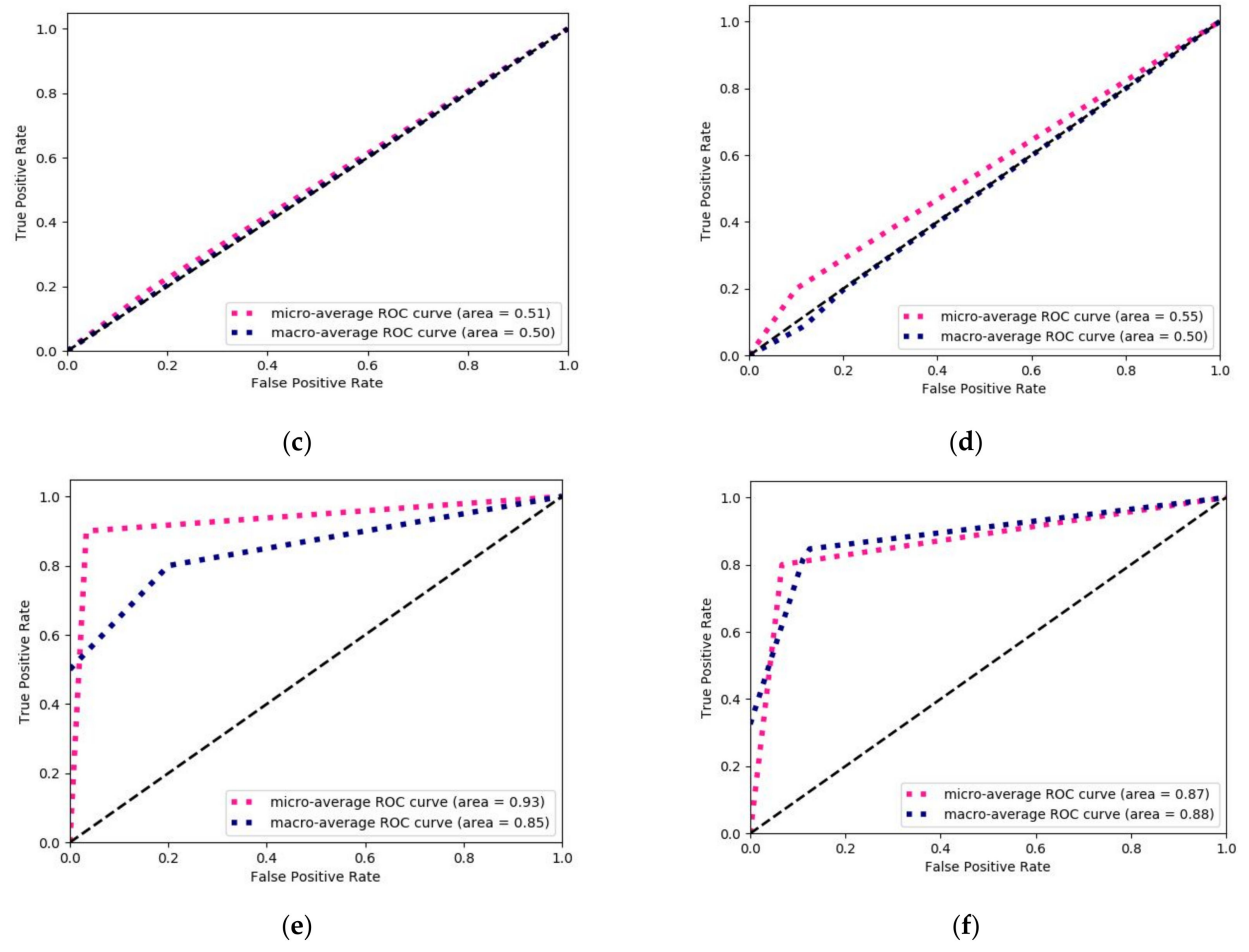
The ROC curves with AUC for different algorithms (**a**) Random Forest, (**b**) Support Vector Machine, (**c**) Logistic Regression, (**d**) Multilayer Perceptron, (**e**) K-Nearest Neighbors, and (**f**) AdaBoost, for tumor staging taking TAS as a prognostic factor.

In addition to the different evaluation metrics, the ROC curve with the area under the curve was also plotted for determining the diagnostic ability of the models. However, the ROC curve is best suitable for the binary classification problem. Therefore, using the Scikit learn, we plotted the ROC curve for multiclass classification by binarizing the output produced for each label or class. In case of multiclass classification, the evaluation measure includes “micro-average” and “macro-average”, where macro-average gives equal weight to the classification of each label. The outputs obtained for each ML algorithm considering the micro-average and macro-average are shown in Figure 3a–f. It was observed that the macro-average AUC (area) for Random-Forest is 0.94, which was better as compared to other models.

### 3.3. Machine Learning-Based Prediction of DFS Period

**Figure 4:**
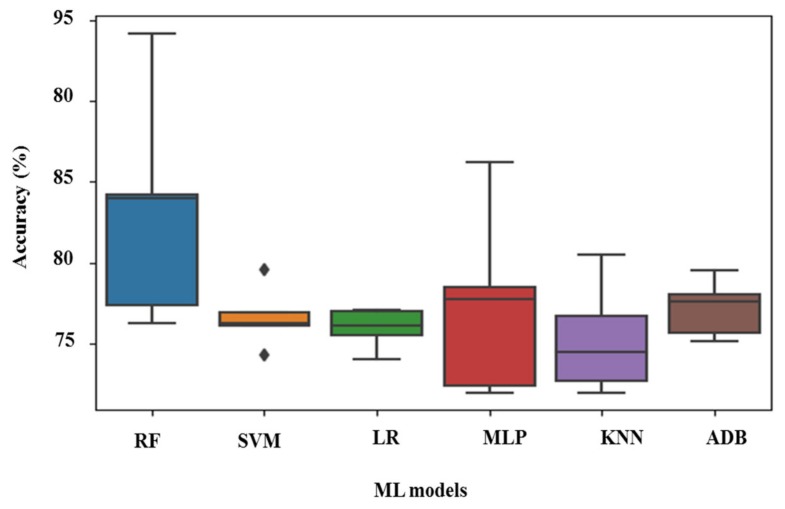
Overall accuracy achieved for predicting the five years disease-free survival.

In addition to the prediction of the tumor stages of the colon cancer, we were also curious to use the TAS for predicting the five years DFS of the colon cancer patients by using the Random Forest, SVM, Logistic Regression, MLP, KNN, and AdaBoost classifiers as the prediction models. The results obtained from different models were shown in Figure 4, where it was observed that the Random Forest outperformed over other algorithms with an accuracy of approximately 84%.

The Table 7 and Table 8 show the values of various evaluation metrics achieved by different ML models using training models and finally model evaluation using the testing models. As seen from the Table 7 and Table 8, Random Forest achieved highest accuracy during the evaluation, which was 0.76 and F-measure was 0.71.

**Figure 5:**
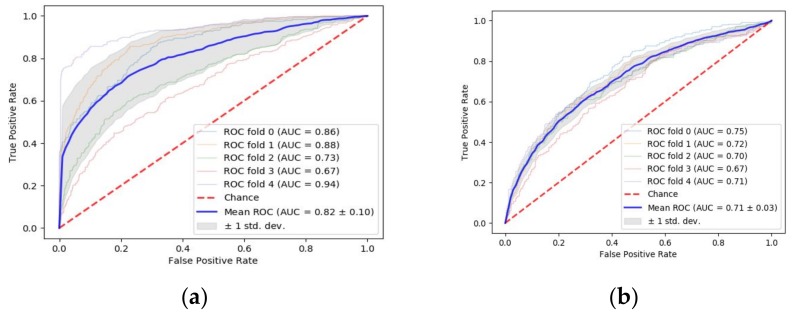

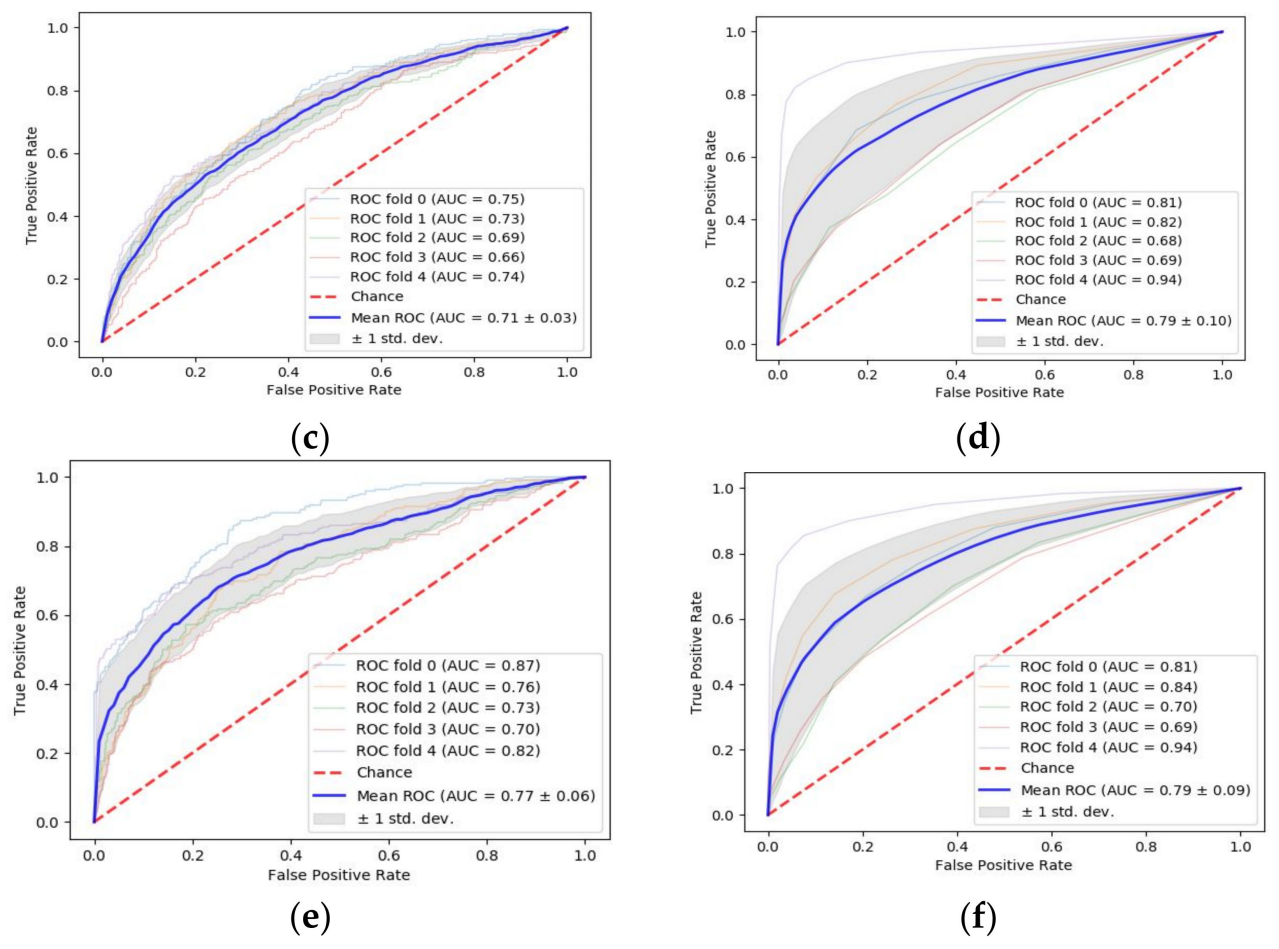
The ROC curves with AUC for different algorithms (**a**) Random Forest, (**b**) Support Vector Machine, (**c**) Logistic Regression, (**d**) Multilayer Perceptron, (**e**) K-Nearest Neighbors, and (**f**) AdaBoost, for predicting the five years DFS of the colon cancer patients.

In addition to the accuracy achieved by different ML algorithms, we also plotted the AUC for each algorithm to see how much the model is able to distinguish between the binary classes of DFS ≥5 years and DFS <5 years. The experiment was carried out using five-fold cross-validation, results of which are shown in Figure 5.

As shown in Figure 5, it was found that the Random Forest achieved AUC of 0.82 ± 0.10 and the second top-performing algorithms were MLP and AdaBoost with AUC of 0.79 ± 0.10 and 0.79 ± 0.09, respectively.

## 4. Discussion

**Figure 6:**
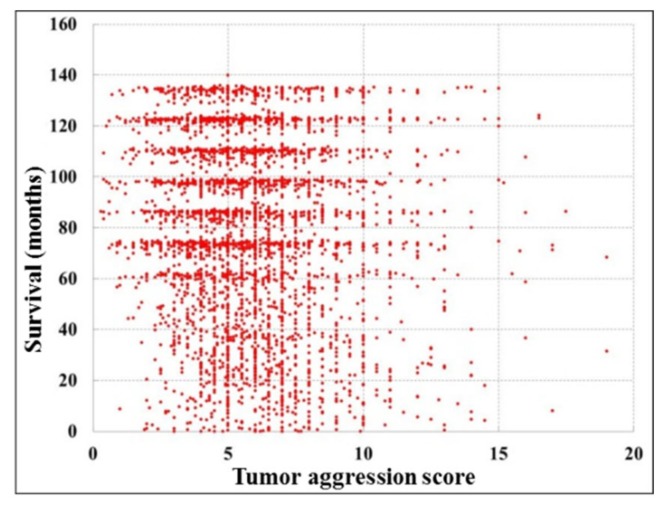
DFS of patients with an increase in Tumor Aggression Score.

It is to be noted that 3709 patients had TAS <9.8 with higher DFS period, which is usually more than five years (60 months) among all the patients considered in this study. The patients were divided into sub-groups considering the median of the TAS. Moreover, it was observed from the Figure 6 that there were patients with DFS >10 years (120 months) in case of TAS <9.8. However, there were a few survival cases found with TAS ≥9.8. The influential parameters such as the presence of circumferential involvement was more in the case of TAS ≥9.8, 91.16% as compared to 8.84% showing the absence when classified into two groups as shown in Table 2. Similarly, the Grade III tumor differentiation was 35.37% in case of TAS ≥9.8, which was more as compared to 6.22% in case of TAS <9.8. When the tumor length exceeded from 4.4 cm, it was found that 96.6% of the patients had TAS ≥9.8. Similarly, when the tumor width exceeded from 4.4 cm, we observed that 97.27% of the patients had TAS ≥9.8. It was observed from the Figure 6 that the number of patients with DFS ≥5 years was greater than the number of patients with DFS <5 years.

There were several studies, where authors considered the tumor size as a prognostic factor for predicting the stages of the colon cancer. In a study of 1734 numbers of T4bN0-2M0 colon cancer patients [29], authors found that smaller size of resected tumor was independent prognostic factor associated with the poorer cancer-specific survival (CSS) in the T4bN0-2M0 with *p* = 0.024 for T4bN0 patients, and a trend of association in T4bN1 with *p* = 0.182 and T4bN2 patients with *p* = 0.191. The five year CSS was 58.4% for the patients with tumor size ≤4.0 cm, 69.3% for the patients with tumor size 4.0–7.0 cm, and 72.4% in those with tumor size ≥7.0 cm. Patients with tumor size 4.0–7.0 cm were not likely to have a significantly different CSS (HR = 0.790, 95%CI: 0.554–1.127; *p* = 0.194), while patients with tumors ≥7.0 cm were more likely to show higher CSS (HR = 0.656, 95% CI: 0.464–0.926; *p* = 0.017). Considering the ML, authors in [25] have used the ML with random optimization to extract the prognostic information from the electronic health records of the breast cancer patients. The information retrieved through the ML-based decision support system was used to predict the progression-free survival with accuracy of 86%. Another ML-based analysis was discussed in [26], where the importance of the micro RNAs was validated. The feature selection in this study was performed using the Information gain and Chi-Squared methods. Furthermore, the classifications of the cancers using the selected micro RNAs were performed using SVM and Random Forest. The performance of the classifier suggested that using three micro RNAs as biomarkers for the breast cancer detection and diagnosis served same effectiveness as using the entire set of 1800 micro RNAs. Similarly, the use of SVM is discussed in [27] for developing a predictive model for the chemo-response in case of epithelial ovarian cancer patients. It was found that the patients stratified into groups with high responses had good responses and favorable prognosis. On the other hand, patients classified into medium to low response groups underwent other trials and drug treatment.

During our experiments, we observed that the results obtained had poor accuracy when the parameters were not initialized efficiently and hyper-parameters were not declared correctly. In addition, the models took a long time to train, even if using the GPU. The algorithms could not converge in case of improper initialization, resulting in poor performance. Therefore, we used the automatic hyper-parameter optimization method of Scikit-optimize.

Our study had other limitations too, such as the data was related to one population of similar types. Our data were derived from a single center and therefore there was the possibility that certain factors were inherent to the Chang Gung Memorial Hospital, Taiwan, such as that patients’ characteristics and health care providers may not reflect the situation in other hospitals or environments. In addition, we observed during our experiments that different models performed differently with TAS. The performance of the Random Forest was best among all the models with an F-measure of 0.89. However, some of the models did not perform well, which requires further study and will be carried out with more data from different populations.

## 5. Conclusions

In this study, we derived the tumor aggression score as a new prognostic factor for predicting the tumor stage and DFS of the colon cancer patients. Furthermore, we used different ML algorithms for training the system with different cross-validations and evaluated the models using independent test data sets. The use of feature selection within cross-validation validated the robustness of the models, where it was observed that Random Forest performed well with an accuracy of 0.89 for the tumor staging. Furthermore, the top-performing model Random Forest achieved an accuracy of 0.84 and AUC of 0.82 ± 0.10 for predicting the five years DFS of the colon cancer patients. It was also observed that the patients with TAS ≥9.8 had poor DFS, whereas the DFS were found to exceed 10 years of survival in case of patients with TAS <9.8.

In the future, we plan to continue our experiment with more data to evaluate the models and design robust ML based prediction models for predicting the survival of the colon cancer patients based on the predicted tumor stage with TAS as a prognostic factor. We would like to include large cohorts of data and parameters from different populations and hospitals to facilitate the mass-level implementation of ML based colon cancer tumor staging and DFS prediction system.

## Figures and Tables

**Table 1:** Sources of parameters collected in the clinical system. TNM: tumor, node, and metastasis.

Sources	Parameters Collected
**Chart records**	Age, gender, adjuvant therapy, status of follow-up, medical illness, pre-operation lab data
**History taking**	Smoking history, coffee consumption, alcohol consumption, physical activity
**Intra-operative finding**	Operation date, intent of resection, operation timing, operation finding, operation type, early morbidity, late morbidity, mortality
**Histo-pathology** **Reports**	Tumor location, gross appearance, circumferential involvement, tumor size, histologic type, histologic grade, tumor extension, examined lymph node number, total positive lymph node number, TNM staging

**Table 2:** Parameters used in this study with *p*-value.

Parameters	Tumor Aggression Score		*p*-
	<9.8 (3709)	≥9.8 (294)	
BMI			0.004
<18.5	215 (5.8)	35 (11.90)	
18.5–23.9	1665 (44.89)	151 (51.36)	
24.0–26.9	1070 (28.85)	66 (22.45)	
≥27	759 (20.46)	42 (14.29)	
Family History (FH)			<0.001
No	2145 (57.83)	180 (61.23)	
Yes	1429 (38.53)	104 (35.37)	
Unknown	135 (3.64)	10 (3.4)	
Age			0.007
<50	527 (14.20)	50 (17)	
≥50	3182 (85.80)	244 (83)	
Gender			<0.001
Male	2114 (57)	165 (56.12)	
Female	1595 (43)	129 (43.88)	
Hypertension			<0.001
Yes	2447 (65.97)	191 (64.96)	
No	1262 (34.03)	103 (35.04)	
Diabetes			<0.001
Yes	3136 (84.55)	231 (78.57)	
No	573 (15.45)	63 (21.43)	
Smoking			0.001
Never	2324 (62.66)	174 (59.18)	
Ex-Smoker	546 (14.72)	42 (14.29)	
Current	839 (22.62)	78 (26.53)	
Alcohol			<0.001
Never	2622 (70.69)	213 (72.45)	
Ex-Drinker	218 (5.88)	18 (6.12)	
Current	869 (23.43)	63 (21.43)	
CEA Level			<0.001
<5	2424 (65.35)	145 (49.32)	
≥5	1285 (34.65)	149 (50.68)	
Hemoglobin			0.9
Low (<11)	853 (23)	182 (61.90)	
Normal	2856 (77)	112 (38.10)	
LAB_ALB			<0.001
≤3.5	424 (11.43)	128 (43.54)	
˃3.5	3285 (88.57)	166 (56.46)	
LAB_CR			<0.001
≤1.1	2954 (79.64)	233 (79.25)	
˃1.1	755 (20.36)	61 (20.75)	
WBC			<0.001
≤5500	202 (5.5)	14(4.8)	
˃5500	3507 (94.5)	280(95.2)	
OP Time			0.001
Elective	3635 (98)	284 (96.6)	
Emergency	74 (2)	10 (3.4)	
OP Find			<0.001
None	3199 (86.25)	205 (69.73)	
Combined	470 (12.67)	84 (28.57)	
Any one	40 (1.08)	5 (1.7)	
CirInvo			<0.001
No	1972 (53.17)	26 (8.84)	
Yes	1737 (46.83)	268 (91.16)	
Tumor Differentiation			<0.001
Grade I	477 (12.86)	7 (2.38)	
Grade II	3001 (80.91)	183 (62.24)	
Grade III	231 (6.22)	104 (35.37)	
Tumor Width			<0.001
≤4.4	2582 (69.61)	8 (2.73)	
˃4.4	1127 (30.39)	286 (97.27)	
Tumor Length			<0.001
≤4.4	2679 (72.22)	10 (3.4)	
˃4.4	1030 (27.78)	284 (96.6)	
T stage			<0.001
T1	377 (10.16)	5 (1.70)	
T2	531 (14.32)	4 (1.36)	
T3	2322 (62.61)	184 (62.59)	
T4	479 (12.91)	101 (34.35)	
N stage			<0.001
N0	2062 (55.6)	179 (60.89)	
N1	1010 (27.23)	57 (19.39)	
N2	522 (14.07)	46 (1.24)	
N3	115 (3.10)	12 (4.08)	

**Table 3:** Performance of different machine learning (ML) training models for tumor staging taking only the tumor size as a prognostic factor.

Algorithms	Evaluation Metrics (Average(± sd))			
	Accuracy	Precision	Recall	F-Measure
**Random Forest**	0.73 (± 0.01)	0.70 (± 0.03)	0.74 (± 0.01)	0.67 (± 0.01)
**Support Vector Machines**	0.63 (± 0.00)	0.39 (± 0.00)	0.63 (± 0.00)	0.48 (± 0.00)
**Logistic Regression**	0.63 (± 0.00)	0.39 (± 0.00)	0.63 (± 0.00)	0.48 (± 0.00)
**Multilayer Perceptron**	0.63 (± 0.00)	0.44 (± 0.12)	0.63 (± 0.02)	0.48 (± 0.00)
**K-Nearest Neighbor**	0.64 (± 0.01)	0.57 (± 0.01)	0.64 (± 0.01)	0.53 (± 0.02)
**Adaptive Boosting**	0.73 (± 0.01)	0.72 (± 0.08)	0.73 (± 0.01)	0.66 (± 0.01)

**Table 4:** Performance of different ML testing models for tumor staging taking only the tumor size as a prognostic factor.

Algorithms	Evaluation Metrics			
	Accuracy	Precision	Recall	F-Measure
**Random Forest**	0.74	0.77	0.74	0.67
**Support Vector Machines**	0.64	0.47	0.64	0.51
**Logistic Regression**	0.65	0.48	0.65	0.54
**Multilayer Perceptron**	0.67	0.55	0.67	0.58
**K-Nearest Neighbor**	0.63	0.50	0.63	0.51
**Adaptive Boosting**	0.67	0.54	0.67	0.57

**Table 5:** Performance of different ML training models for tumor staging taking TAS as a prognostic factor.

Algorithms	Evaluation Metrics (Average (± sd))			
	Accuracy	Precision	Recall	F-Measure
**Random Forest**	0.90 (± 0.01)	0.90 (± 0.02)	0.90 (± 0.02)	0.90 (± 0.02)
**Support Vector Machines**	0.73 (± 0.02)	0.58 (± 0.08)	0.73 (± 0.02)	0.63 (± 0.02)
**Logistic Regression**	0.63 (± 0.00)	0.41 (± 0.00)	0.63 (± 0.00)	0.49 (± 0.00)
**Multilayer Perceptron**	0.63 (± 0.02)	0.41 (± 0.07)	0.63 (± 0.02)	0.50 (± 0.03)
**K-Nearest Neighbor**	0.86 (± 0.01)	0.88 (± 0.01)	0.86 (± 0.01)	0.85 (± 0.01)
**Adaptive Boosting**	0.89 (± 0.01)	0.89 (± 0.01)	0.89 (± 0.01)	0.89 (± 0.01)

**Table 6:** Performance of different ML testing models for tumor staging taking Tumor Aggression Score (TAS) as a prognostic factor.

Algorithms	Evaluation Metrics			
	Accuracy	Precision	Recall	F-Measure
**Random Forest**	0.89	0.89	0.88	0.89
**Support Vector Machines**	0.73	0.65	0.73	0.64
**Logistic Regression**	0.62	0.38	0.62	0.48
**Multilayer Perceptron**	0.62	0.52	0.64	0.48
**K-Nearest Neighbor**	0.85	0.87	0.85	0.84
**Adaptive Boosting**	0.81	0.81	0.81	0.78

**Table 7:** Performance of different ML training models for predicting the five years disease-free survival (DFS).

Algorithms	Evaluation Metrics (Average (± sd))			
	Accuracy	Precision	Recall	F-Measure
**Random Forest**	0.84 (± 0.12)	0.82 (± 0.14)	0.83 (± 0.12)	0.81 (± 0.14)
**Support Vector Machines**	0.77 (± 0.03)	0.74 (± 0.07)	0.77 (± 0.03)	0.71 (± 0.05)
**Logistic Regression**	0.76 (± 0.02)	0.73 (± 0.04)	0.76 (± 0.02)	0.71 (± 0.02)
**Multilayer Perceptron**	0.78 (± 0.11)	0.77 (± 0.10)	0.77 (± 0.11)	0.77 (± 0.12)
**K-Nearest Neighbor**	0.75 (± 0.06)	0.72 (± 0.08)	0.75 (± 0.06)	0.71 (± 0.02)
**Adaptive Boosting**	0.77 (± 0.03)	0.75 (± 0.04)	0.77 (± 0.03)	0.74 (± 0.03)

**Table 8:** Performance of different ML testing models for predicting the five years DFS.

Algorithms	Evaluation Metrics			
	Accuracy	Precision	Recall	F-Measure
**Random Forest**	0.76	0.74	0.76	0.71
**Support Vector Machines**	0.74	0.71	0.74	0.64
**Logistic Regression**	0.73	0.70	0.73	0.71
**Multilayer Perceptron**	0.64	0.66	0.64	0.65
**K-Nearest Neighbor**	0.73	0.70	0.73	0.70
**Adaptive Boosting**	0.66	0.70	0.66	0.67
